# Entering and exiting behaviour of the phlebotomine sand fly *Lutzomyia longiflocosa* (Diptera: Psychodidae) in rural houses of the sub-Andean region of Colombia

**DOI:** 10.1590/0074-02760160265

**Published:** 2016-12-01

**Authors:** Raúl Hernando Pardo, Erika Santamaría, Olga Lucia Cabrera

**Affiliations:** 1 Instituto Nacional de Salud, Grupo de Entomología, Bogotá, Colombia; 2 Universidad de La Salle, Grupo de Entomología y Enfermedades Transmitidas por Vectores, Bogotá, Colombia

**Keywords:** host seeking behaviour, house entry, Lutzomyia, sand flies

## Abstract

The present study identified the entering and exiting sites for *Lutzomyia longiflocosa* in rural houses of the sub-Andean region in Colombia. Entering sites were identified with sticky traps set up outside the bedrooms, around the eave openings, and with cage traps enclosing the slits in the doors and windows inside the bedrooms. Exiting sites were identified by releasing groups of females indoors. These females were blood fed and marked with fluorescent powders. Females were recaptured with the trap placement described above but set up on the opposite sides of the openings. In the entering experiment, a significantly higher number of females were captured in the sticky traps at the zone nearest the eave openings (n = 142) than those captured in the other zones of the trap (n = 52); similarly, a higher number of females were captured on the front side of the house (n = 105) than at the rear side (n = 37). Only two females were collected in the cage trap. In the exiting experiment, at the ceiling, the highest percentage (86.2%) of females was recaptured with sticky traps nearest the eave openings and on the front side of the house (70.0%). Seven females were collected in the cage trap. *Lu. longiflocosa* entered and exited houses primarily through the eave openings in a non-random pattern in relation to the sides of the house.

Phlebotomine (subfamily Phlebotominae) females are vectors of the aetiological agents of at least six diseases (Maroli et al. 2013), among which leishmaniasis, caused by parasites of the genus *Leishmania*, is the most common. Cutaneous leishmaniasis (CL) is the most dominant form of leishmaniasis in the Andean region. In this region, Colombia historically records the highest number of reported cases of CL (Davies et al. 2000, WHO 2016). The average annual number of cases recorded between 2011 and 2015 was approximately 9,500 (SIVIGILA/INS 2016) and mostly originating in the sub-Andean region between 1,100 and 2,400 m asl. Among the seven phlebotomine species known to act as vectors of five *Leishmania* species in Colombia, *Lutzomyia longiflocosa* Osorno-Mesa, Morales, Osorno & Hoyos 1970, is considered as the main vector in the largest epidemics of CL (caused by *L. braziliensis* and *L. guyanensis*) recorded in the country (Morales et al. 2004, Pardo 2006). *Lu. longiflocosa* is an endemic species of the sub-Andean region with anthropophagic and endophagic behaviour, reaching densities of up to 50 females/CDC trap/night indoors (Pardo 2006). These observations indicated that disease transmission typically occurs indoors and in its nearest surroundings, as has been shown elsewhere in the Andean region (Davies et al. 2000).

The indoor behaviour of endophagic phlebotomine females is part of their gonotrophic cycle, which is defined as the sequence of behavioural and physiological phases in the female that begins with host seeking and ends with egg laying. Based on the definitions of the phases of host seeking behaviour in mosquitoes (Clements 1999) and host seeking and post-feeding behaviour in hematophagous insects (Lazzari 2009), the behaviour of phlebotomine females indoors and in surrounding areas can be expected to include the following three phases of the cycle: (1) host seeking (close range seeking), (2) attack, and (3) host leaving (initial host leaving). The behavioural events that occur during these phases are unknown or rarely studied, and their study is difficult. The difficulties related to the studies of phlebotomine female indoor behaviour under field conditions are similar to those of mosquito studies (Cardé & Gibson 2010). The difficulties include the following factors: (1) the small size of phlebotomine sand flies, with a linear dimension three times smaller than that of mosquitoes, (2) the predominantly nocturnal host seeking behaviour, and (3) the females can migrate relatively large distances of up to a few hundreds of meters during the entire host seeking phase. The entering and exiting behaviour of phlebotomine females belongs to the close range host seeking and the initial host leaving phases of the gonotrophic cycle, respectively. Although the particular mechanism of host location is unknown, the Phlebotominae probably locates the host as weak flying insects do by flying upwind until they locate a host odour plume (Ready 2013). During close range host seeking, carbon dioxide and human kairomones can play dominant roles in female orientation. A field study showed that *Lu. intermedia* and *Lu. whitmani* females were attracted to traps baited with carbon dioxide, and were attracted in even greater numbers to traps baited with human kairomones (Pinto et al. 2001). When females arrive at the outer structure of a house, they must choose to enter through several available points of entry. It is presumed that females enter houses mainly through large openings (*e.g.*, doors, windows, and eaves), although small cracks in walls and ceilings may be equally important entry points (Alexander et al. 1995). Nevertheless, few published studies address this subject. In Marajo Island, Brazil, a study on the determinants of the indoor abundance of sand flies found a significant positive association between the indoor density of *Lu. longipalpis* and the size (qualitatively measured) of the house openings (Quinell & Dye 1994a). The preference for the location of the openings was not evaluated. After blood-feeding, females search a resting site either indoors (endophily) or outdoors (exophily) to digest the blood and develop the eggs. Although sand fly females are assumed to leave through the same openings that were used to enter the house, this behaviour has not been documented. Variations in this behaviour may be expected to occur due to limitation in the mobility of blood-fed females, which increased approximately two times their original weight after a blood meal.

No detailed studies have been currently performed to assess the entering and exiting behaviour of phlebotomine females. Most knowledge of the entering and exiting behaviour of the anthropophilic and endophagic Diptera comes from mosquitoes using experimental huts where, interception traps (mainly cage traps with or without entry slots) are used to catch host-seeking females, as well as females that are exiting a house through windows, doors, slits on walls, and eaves. Infrared videos have been used recently to describe the house entry behaviour of *Anopheles gambiae* under semi-field conditions (Spitzen et al. 2016). In the present study performed in houses in the sub-Andean region, the openings can be classified as either small or large. Small openings may be sampled with the cage trap used in the experimental huts for mosquitoes. Large openings, such as those in open eaves, may be sampled by sticky traps which are easy to fit and inexpensive. The target eave opening may be partially blocked by sticky traps to reduce the possible effect of interception trap on flight paths.

Identification of how endophagic sand flies enter and exit a house is important in understanding host seeking and post-blood-feeding behaviour. This knowledge will allow a better comprehension of the impact of indoor vector control measures and the development of new control methods. The present study aimed to describe the exiting and entering behaviour of *Lu. longiflocosa* females under field conditions in rural houses of sub-Andean Colombia; this study focused on the preferences in size and location of the openings used by females. Comparison of abundance (entrance sites) and percentages of recapture (exit sites) females were performed using sticky traps around eaves and cage traps enclosing slits.

## MATERIALS AND METHODS

*Study area* - The study was performed in the Colombian state of Huila, Campoalegre County in the dispersed rural settlement of Venecia. The settlement centre was located at 2º39′47″N and 75º14′31″W. The annual precipitation in this area is approximately 1,000 mm, which occurs bimodally with the low rainfall periods in the first two months of the year and again at mid-year. The average daily temperature is 20ºC. This is a rural area with an economy based entirely on unshaded coffee production. The experiments were completed during periods of low precipitation; these present the highest risk seasons for phlebotomine sand flies when *Lu. longiflocosa* occurs in pronounced peaks of abundance (Carvajal 2008). During these periods, phlebotomine sand fly abundance increases to up to 250 females/CDC trap/night outdoors (forest) and over 50 females/CDC trap/night indoors. In contrast, during high precipitation periods, phlebotomine sand fly abundance decreases drastically both indoors and outdoors to between 0 and 5 females/CDC trap/night, thus making it difficult to carry out any behavioural study (Carvajal 2008). Three houses were selected with similar construction styles and a history of high levels of phlebotomine female infestation. The houses were located at an elevation of approximately 1,600 m and separated by distances between 500 and 1,500 m. During the night-time collection periods, *i.e.*, between 18 h and 06 h, no precipitation was recorded, and the mean temperature and relative humidity were 19.3ºC and 70%, respectively.

*Description and adaptations of houses for the experiment* - Each selected house had two or three bedrooms, a single door, and two windows. The walls were constructed using adobe or brick, and the houses had cement flooring and a roof of galvanised sheet metal. The latter was extended to form two eaves, one forming a porch-like extension in the front of the house and the second forming a projection to the rear. Two of the houses had its bedrooms with ceilings made of boards with open slits between the boards. Each house had two kinds of openings to the exterior, generally rectangular, with the following standard form: (1) large eave gaps located at 2.2 m above floor level and between the upper edge of the wall and the roof with a width of 10 cm and a median area of 1.6 m^2^ and (2) narrow slits between the edges of the door and windows, several millimetres wide with a median area of 0.3 m^2^/house. Open windows and doors also provided large openings, but during the present study, they customarily remained closed at night. This was done for security reasons, and because the temperature in the sub-Andean region has strong daily fluctuations, and night-time temperature is considered cold. On average, eight persons inhabited each house.

In each house, a bedroom was selected that had an exterior door (one of the three had a window as well). The eave gaps of each room had an average (median) width of 10 cm with an average area of 0.7 m^2^. The narrow slits provided openings with an average width of 1.0 cm and an average area of 0.05 m^2^. During the experiment, the usual bedroom occupants were moved to another bedroom in the same house where they remained asleep until the experiment was finished. Some furniture was removed to facilitate the observation of phlebotomine sand flies and trap placement. The bedrooms were not airtight, because they had openings between the ceiling boards or they had no ceiling; hence, recapture of phlebotomine sand flies was difficult. Therefore, in each modified bedroom, a white plastic canvas sheet was placed to form the ceiling. The openings connecting with the other rooms were blocked with plastic sheeting as well, so that the only apertures were those openings to the outside of the house. This reduced the possible interference on the phlebotomine sand fly collections from people sleeping in other bedrooms. Lastly, the floor was covered with white plastic to assist in the recovery of fallen phlebotomine sand flies.

**Fig. 1:**
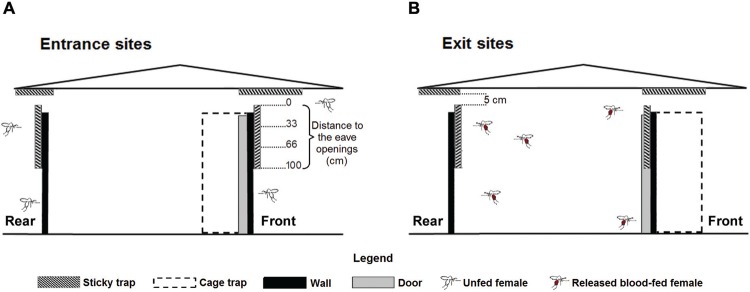
: general layout of the sticky traps and cage traps in the modified bedroom of a house during the entrance and exit site experiments. Drawing not to scale (see text for details).

*Identification of entrance sites: capture of phlebotomine sand flies* - Two methods of phlebotomine sand fly capture were used depending on the aperture type evaluated. Phlebotomine sand flies attempting to enter through the eave openings were captured by sticky traps. The traps consisted of sheets of bond paper, 100 x 70 cm (area = 10.7 m^2^/modified bedroom), impregnated with castor bean oil. The traps were placed to the exterior of the modified bedroom on the walls and ceiling over the eave openings (Fig. 1A). In order for the sticky traps to intercept the largest number of females and to reduce the possible effect of this interception trap on flight paths, traps on the walls were placed at a distance of 5 cm from the raised ceiling. The assumption was that the females captured nearest the openings attempted to pass through them. Therefore, the distance between the opening and the location of the captured phlebotomine sand flies was divided into three sections: 0-33 cm (intention of passing), 33-66 cm, and 66-100 cm (Fig. 1A). A recent study on *An. gambiae* confirmed the assumption that endophagic Diptera observed nearest the eave openings attempt to pass through them (Spitzen et al. 2016). The study showed that *An. gambiae* that pass through the eaves spend most of the observed time within 30 cm of the eave. Phlebotomine sand flies attempting to enter between the slits in the doors and windows were captured by cage trap using a modified Muirhead-Thomson trap (Muirhead-Thomson 1947). The trap consisted of a box in the form of a rectangular prism with the 2 bases and 3 sides made of cloth and the remaining side left open. The trap was placed inside the modified bedroom such that it enclosed the open slits around the doors and windows (Fig. 1A).

*Experimental design* - The purpose was to compare the numbers of field phlebotomine females entering a modified bedroom through two potential entry points: (1) through the large eave gaps by sticky traps and (2) through narrow slits in the doors and windows using the cage trap. The phlebotomine sand flies were removed from the traps each half-hour for 10 min between 21 h and 01 h. For the experimental operation, three volunteers participated in the following three formats: (a) one volunteer stayed in the interior of the modified bedroom recovering flies trapped in the cage traps over the door/window slits and those in the first 20 cm of the sticky traps at the ceiling near the eave openings in the front and rear of the house; (b) a second volunteer, situated outside the bedroom, removed flies from the sticky traps located on the external walls and adjacent to the ceiling near the eave openings at the front and rear of the house; (c) the third volunteer was situated in the bedroom centre serving as human bait (protected) to evaluate the efficacy of the sticky trap. Trap efficiency referred to the ability to collect most of the flies without completely closing off the bedroom openings. The observations were taken in two consecutive nights in each of the three houses. The first house was sampled in March, another in July, and the last in August 2011 (n = 6). Data recorded were as follows: (a) number of female *Lu. longiflocosa* attempting to enter through the two types of openings, (b) on the sticky traps, the distance from insect to opening was categorised, and (c) the side of the house, front or rear, where the fly was captured.

*Identification of exit sites:* *capture of phlebotomine females* - The same openings to the exterior tested in the experiment on the entrance sites were examined for the identification of the probable exit points. This was tested using the same traps as the entry collection with several modifications. For the eave openings, sticky traps were placed on the interior walls of the modified bedrooms, whereas the sticky traps on the ceiling remained outside, the same place in the experiment on the entrance sites (Fig. 1B). The latter were not installed inside for two reasons: (a) the precarious structure of the ceiling made placement of the sticky traps difficult, and (b) this placement presented inconvenience for the inhabitants. In sticky traps located on the external ceiling, the interpretation of the attempt to pass of the collected phlebotomine females was different from that collected by sticky traps on the walls. The flies collected more distant from an opening (33-66 cm and 66-100 cm) indicated those that had actually passed through the opening, whereas those nearer the opening (0-33 cm) indicated flies with the probable intent of passing in through the nearby opening. The cage traps around the slits of the doors and windows were placed outside (Fig. 1B).

*Experimental design* - Field females of *Lu. longiflocosa* were marked with fluorescent powder (Pardo et al. 1996), blood fed to repletion on an anesthetised golden hamster (*Mesocricetus auratus*), and released from the centre of the experimental bedroom. To reduce damage to the phlebotomine females by handling and to allow their rapid release, the flies were fed in a cage with folding sides and then opened mechanically for release of the flies into the modified room. The blood-fed flies served two purposes. First, the released flies did not bite the volunteers or the other sleeping inhabitants of the house. Second, the flies provided information on the post-feeding behaviour to compare with those after feeding on humans. Finally, to avoid experimental interference by the attraction of host seeking field phlebotomine females to the volunteers, the exterior front and rear of the modified bedroom were enclosed with fine mesh mosquito net. The number of marked females that were recaptured was then recorded, along with the type of opening, the distance to the opening, and the exiting position. The roles of the three volunteers in recapturing the phlebotomine females were as follows: (a) the first, situated inside the modified bedroom, recorded the flies recovered from the sticky traps, (b) the second, situated outside the modified bedroom, recorded the flies in the cage and sticky traps outside the ceiling, as well as those that avoided both and were entrapped in the mosquito net enclosing the bedroom, and (c) the third, situated in the centre of the bedroom, acted as human bait to verify that the released flies were unable to feed. The trial in each house was carried out immediately after the set of entrance observations with the same duration, intervals, and repetitions (n = 6).

*Observations common to both experimental sets* - Upon completion of the phlebotomine sand fly entrance and exit trials, the cage traps were disassembled, and a search was conducted for females that remained resting in the modified bedroom. The volunteers remained in the bedroom until 06 h of the following morning when, again, the sticky traps were inspected, and the walls checked for resting females. The flies were removed from the sticky traps with a paintbrush, placed in a soap solution to remove the oil, and then preserved in 70% ethanol for later laboratory identification.

*Statistical analysis* - Sticky trap density data from the entrance site experiments did not show a normal distribution, but both the 4 h experimental collections and the all-night collections were congruent using the geometric mean of the fly number per square meter (females/m^2^/4 h and females/m^2^/night, respectively) with their 95% confidence interval (95% CI). The capture data from the all-night sticky traps confirmed that the pattern of behaviour observed at the eave openings during the experimental periods continued throughout the night. The cage traps were not operated through the night, because they blocked the exit doorway and thus inconvenienced the inhabitants of the study houses. Fly densities and distances from the openings were compared using analysis of variance (ANOVA). The Generalized Linear Interactive Modelling (GLIM) component was used and assumed a normal distribution of error for the response variable, *i.e.*, the natural log transform of female density [logₑ(female number + 1)]. The model was validated by the quantile-quantile graphic of the residuals against the values predicted and of the observed values against the predicted values. Fly densities at the front and rear sides of the house were compared with the Wilcoxon non-parametric test.

The data for identifying the exit sites were presented as the number and percentage of recaptured flies per trial and in total. The analysis was based on the total percentage of recaptured females. The Chi-square test for polytomic variables was applied in the percentage of females recaptured in the distance-from-opening comparisons. In testing the percentage of recapture between the front and rear of the house, the Chi-square test for single proportions was used. These analyses were available in the Stata© program set, Stata Corporation, version 12.1, and Microsoft Office Excel 2013©.

*Ethics* - For the houses where the observations were made, the head of the household read and voluntarily signed a standard informed consent form. The volunteers, all of whom have had experience with phlebotomine sand fly collection methods and who have participated as human bait, also signed an informed consent form. The use of the golden hamster as a blood source for the released phlebotomine females was approved by the Ethics Committee of the National Institute of Health of Colombia (approved by agreement no. 5, Jun 25, 2009).

## RESULTS

*Entrance sites* - Of the 220 phlebotomine sand flies collected within the modified bedrooms during the 21-01 h period, 218 (99.1%) were *Lu. longiflocosa*, predominantly female (91.7%), and two were females of the subgenus *Helcocyrtomyia*. Almost all the *Lu. longiflocosa* were collected on the sticky traps (212 out of 218, 97.3%) (Table I). Only two flies (female) were captured using the cage traps, and four additional females were attracted to the protected human. Throughout the night (21-06 h), this pattern was maintained. The predominant species was *Lu. longiflocosa* (268/270, 99.2%), and the majority (98%) were unfed females. Only five females had blood fed collected on sticky traps in the 0-33 cm zone of the ceiling. One additional male was collected resting on the modified bedroom wall. Moreover, since the majority of the *Lu. longiflocosa* were females (194 out of 218, 89.0%) collected on the sticky traps during the trial period and all night (231 out of 268, 86.2%), the statistical analyses were applied only to females collected by this method.

**TABLE I:** Capture locations of *Lutzomyia longiflocosa* entering the houses, as collected by sticky traps (placed around the large eave openings) and cage traps (placed around the slits on the doors and windows). Observations were made in three houses on two consecutive nights

Trap type	Side of house	Trap placement	Distance from opening cm	Number of phlebotomine sand flies													

				Experimental period 21-01 h					Post-experimental period 01-06 h					Night total 21-06 h			
		
				♀	♂	total	(%)		♀	♂	total	(%)		♀	♂	total	(%)
Sticky trap	Front	Ceiling	0 - 33	67^a^	9	76	(28.9)		11	4	15	(5.7)		78	13	91	(34.6)
			33 - 66	14	0	14	(5.3)		0	0	0	-		14	0	14	(5.3)
			66 - 100	13	1	14	(5.3)		7	1	8	(3.0)		20	2	22	(8.3)
		Wall	0 - 33	38	5	43	(16.3)		3	1	4	(1.5)		41	6	47	(17.9)
			33 - 66	10	0	10	(3.8)		2	2	4	(1.5)		12	2	14	(5.3)
			66 - 100	13	0	13	(4.9)		2	2	4	(1.5)		15	2	17	(6.5)

		Subtotal		155	15	170	(64.6)		25	10	35	(13.3)		180	25	205	(77.9)
	
	Rear	Ceiling	0 - 33	33^b^	3	36	(13.7)		1	0	1	(0.4)		34	3	37	(14.1)
			33 - 66	0	0	0	-		1	0	1	(0.4)		1	0	1	(0.4)
			66 - 100	1	0	1	(0.4)		1	0	1	(0.4)		2	0	2	(0.8)
		Wall	0 - 33	4	0	4	(1.5)		7	1	8	(3)		11	1	12	(4.6)
			33 - 66	1	0	1	(0.4)		1	1	2	(0.8)		2	1	3	(1.1)
			66 - 100	0	0	0	-		1	0	1	(0.4)		1	0	1	(0.4)

		Subtotal		39	3	42	(16)		12	2	14	(5.3)		51	5	56	(21.3)

	Subtotal			194	18	212	(80.6)		37	12	49	(18.6)		231	30	261	(99.2)
	
Cage trap	Front	Door	NA^c^	2	0	2	(0.8)		NA	NA	NA	-		2	0	2	(0.8)
	Rear	Window	NA	0	0	-	-		NA	NA	NA	-		-	-	-	-

	Subtotal			2	0	2	(0.8)		-	-	-	-		2	0	2	(0.8)

Total				196	18	214	(81.4)		37	12	49	(18.6)		233	30	263	(100)

*a*: three blood-fed females; *b*: two blood-fed females; *c*: not applicable.

**Fig. 2:**
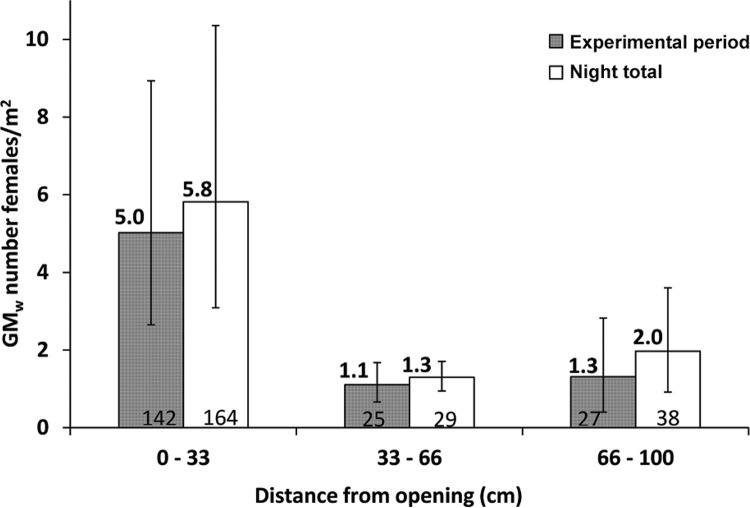
: density of female *Lutzomyia longiflocosa* (geometric mean of Williams, GMW) captured by sticky traps during the trial period (21-01 h) and all night (21-06 h), demarcated by distance from the large eave openings. Error bars show 95% confidence intervals (95% CI). The total numbers of females are shown at the base of the bars.

*Female entry through the large eave openings* - During the 4 h experimental period, the traps nearest the opening (0-33 cm) collected approximately five times, 5.0 females/m^2^/4 h, the number found in the intermediate zone (33-66 cm) or the far zone (66-100 cm) (Fig. 2). The differences among the zones were highly significant (*F*_(2,15)_ = 11.95, p < 0.001). The density of the zone nearest the opening was significantly higher than each of the other two (*z* = -4.42, p < 0.001 and *z* = -4.03, p < 0.001, respectively). For the all-night captures, the results were very similar. The nearest opening collected at least three times, 5.8 females/m^2^/night, the number found in the other two zones (Fig. 2). The ANOVA was highly significant (*F*_(2,15)_ = 13.37, p < 0.001), as were the differences in density between the nearest and the other two zones (*z* = 4.95, p < 0.001 and *z* = 3.78, p < 0.001).

*Front versus rear house entry* - During the 4 h experimental period and using only data from the 0-33 cm zone nearest the large eave openings, the density (95% CI) at the front of the house was approximately four times, 3.0 (2.0-4.3) females/m^2^/4 h (n = 105), that of the rear of the house, 0.8 (0-2.3) females/m^2^/4 h (n = 37). This difference was statistically significant (*z* = 2.20, p = 0.028). For the all-night captures, a similar pattern was observed. With a fly density of 3.4 (2.3-4.9) females/m^2^/night (n = 119) in the 0-33 cm zone from the front of the house and 0.9 (0-2.8) females/m^2^/night (n = 45) from the rear side, this difference was statistically significant (*z* = 1.99, p = 0.005). The combined data from the three sticky trap zones (0-100 cm) during the experimental period also showed the same pattern, with the traps at the front of the house capturing 6 times the number of females captured at the rear side [4.5 females/m^2^/4 h (n = 155) vs. 0.8 females/m^2^/4 h (n = 39); *z* = -2.20, p = 0.028]. This trend continued for the entire night, with 5.3 females/m^2^/night (n = 180) at the front of the house and one female/m^2^/night (n = 51) at the rear of the house (*z* = -2.20, p = 0.028).

*Ceiling versus wall* - During the experimental period, the phlebotomine female density at the ceiling was approximately twice, 3.4 females/m^2^/4 h, that of the wall, 1.9 females/m^2^/4 h; however, the difference was not significant (*z* = 1.57, p = 0.116). For the all-night captures, a similar pattern was observed. Sand fly density of 4.0 females/m^2^/night in the ceiling and 2.5 females/m^2^/night on the wall was also not significant. Nevertheless, significant differences were observed when percentages of phlebotomine females were compared. During the experimental period, a higher number of females were collected (128, 66.0%) at the traps at the ceiling than those at the walls (*z* = 4.43, p < 0.001). The all-night data followed the same pattern where the females recovered (149, 64.5%) were greater at the ceiling than at the walls (*z* = 4.41, p < 0.001) (Table I).

*Exit sites* - A total of 413 phlebotomine females were dusted with fluorescent powder, fed on hamster, and released in 6 trials with an average of approximately 69.2 phlebotomine females per trial. Some phlebotomine female mortality was associated with each release (n = 38, 9.2%), and therefore, 375 survived the trials. During the 4 h experimental period, 177 (47.2%) phlebotomine females were recovered; from this, 96% (170 females) were collected by means of sticky traps and only seven females by cage traps. None were recaptured by attraction to the human bait. When the recapture period was extended to 06 h (all night), 28 additional phlebotomine females were collected from the sticky traps, increasing the total to 205 out of 375 (54.7%) (Table II). Nine additional marked females were found resting elsewhere, but the analytic comparisons were limited to the sticky trap data, since these phlebotomine females directly demonstrated the exit points.

**TABLE II:** Recapture locations of marked blood-fed *Lutzomyia longiflocosa* exiting the houses, as collected by sticky traps (placed around the large eave openings) and cage traps (placed around the slits on the doors and windows). A total of 375 marked and blood-fed females were released in three houses on two consecutive nights

Trap type	Side of house	Trap placement	Distance from opening cm	Number of phlebotomine females							

				Experimental period 21-01 h			Post-experimental period 01-06 h			Night total 21-06 h	
		
				Nº	(%)		Nº	(%)		Total	(%)
Sticky trap	Front	Ceiling	0 - 33	18	(8.8)		5	(2.4)		23	(11.2)
			33 - 66	3	(1.5)		2	(1.0)		5	(2.4)
			66 - 100	1	(1.7)		2	(1.0)		3	(1.5)
		Wall	0 - 33	30	(14.6)		10	(4.9)		40	(19.5)
			33 - 66	20	(9.8)		0	-		20	(9.8)
			66 - 100	40	(19.5)		1	(0.5)		41	(20.0)

		Subtotal		112	(54.6)		20	(9.8)		132	(64.4)
	
	Rear	Ceiling	0 - 33	7	(3.4)		4	(2.0)		11	(5.4)
			33 - 66	0	-		1	(0.5)		1	(0.5)
			66 - 100	0	-		0	-		0	-
		Wall	0 - 33	14	(6.8)		2	(1.0)		16	(7.8)
			33 - 66	9	(4.4)		1	(0.5)		10	(4.9)
			66 - 100	28	(13.7)		0			28	(13.7)

		Subtotal		58	(28.3)		8	(3.9)		66	(32.2)

	Subtotal			170	(82.9)		28	(13.7)		198	(96.6)
	
Cage trap	Front	Door	NA^a^	7	(3.4)		NA			7	(3.4)
	Rear	Window	NA	0			NA				

	Subtotal			7	(3.4)		0			7	(3.4)

Total				177	(86.3)		28	(13.7)		205	(100)

*a*: not applicable.

*Female exit through the large eave openings* - Similar to the entrance captures, the sticky traps were set to capture the marked females as they exited the house. The flies were grouped by distance from the eave opening. The interpretation differed on whether the trap was located on the outside ceiling or on the inside wall of the experimental bedroom. Therefore, the results are presented separately for each site.

The ceiling site collected most of the females (25, 86.2%) in the nearest opening zone (0-33 cm). At the intermediate zone (33-66 cm), three females more were collected, and only one female was captured at the far zone (66-100 cm) from the opening (Table III). These distances were significantly different (*X* ^2^ = 36.7, d.f.: 2, p < 0.001). The all-night statistics were similar, with 34 females (79.1%) recovered nearest the opening, six females more in the intermediate zone, and three additional flies in the far zone (*X* ^2^ = 40.9, d.f.: 2, p < 0.001).

**TABLE III:** Numbers of marked blood-fed *Lutzomyia longiflocosa* recaptured at each distance from the eave exit openings, according to the placement of the trap, using sticky traps

Trap placement	n	Distance to large openings cm														

		Experimental period 21-01 h								All night 21-06 h						
	
		0-33 (%)		33-66 (%)		66-100 (%)		total		0-33 (%)		33-66 (%)		66-100 (%)		total
Ceiling (outside the modified bedroom)																
	1	2	(40.0)	2	(40.0)	1	(20.0)	5		4	(50.0)	3	(37.5)	1	(12.5)	8
	2	4	(100)	0	(0)	0	(0)	4		6	(60.0)	2	(20.0)	2	(20.0)	10
	3	7	(100)	0	(0)	0	(0)	7		7	(100)	0	(0)	0	(0)	7
	4	1	(100)	0	(0)	0	(0)	1		3	(100)	0	(0)	0	(0)	3
	5	6	(100)	0	(0)	0	(0)	6		6	(100)	0	(0)	0	(0)	6
	6	5	(83.0)	1	(16.7)	0	(0)	6		8	(88.9)	1	(11.1)	0	(0)	9

Total		25 (86.2)		3 (10.2)		1 (3.4)		29		34 (79.1)		6 (14.0)		3 (7.0)		43

Wall (inside the modified bedroom)																
	1	8	(66.7)	3	(25.0)	1	(8.3)	12		8	(66.7)	3	(25.0)	1	(8.3)	12
	2	4	(44.5)	1	(11.0)	4	(44.5)	9		7	(58.3)	1	(8.3)	4	(33.3)	12
	3	6	(25.0)	1	(4.2)	17	(70.8)	24		8	(38.8)	1	(3.8)	17	(65.4)	26
	4	2	(6.3)	8	(25.0)	22	(68.7)	32		4	(11.8)	8	(23.5)	22	(64.7)	34
	5	19	(54.3)	6	(17.1)	10	(28.6)	35		22	(56.4)	6	(15.4)	11	(28.2)	39
	6	5	(17.2)	10	(34.5)	14	(48.3)	29		7	(21.9)	11	(34.4)	14	(43.7)	32

Total		44 (31.2)		29 (20.6)		68 (48.2)		141		56 (36.1)		30 (19.4)		69 (44.5)		155

Inside the modified bedroom walls, most females were recaptured in the far zone from the large eave opening, 68 (48.2%), 44 (31.2%) were captured nearest the opening and 29 (20.6%) in the intermediate zone (Table III). These differences were statistically significant as well (*X* ^2^ = 16.5, d.f.: 2, p < 0.001). The all-night collection numbers again paralleled those of the experimental period at the far (69, 44.5%), nearest (56, 36.1%), and intermediate zones (30, 19.4%) and were significantly different (*X* ^2^ = 15.3, d.f.: 2, p = 0.002) (Table III).

*Front versus rear house exit* - During the experimental period, the number of females collected (48, 69.6%) at the traps closest to the eave openings (0-33 cm) at the front of the house was significantly higher than that at the rear side (*X* ^2^ = 10.6, d.f.: 1, p = 0.001). When the all-night data were evaluated, the same pattern was observed, *i.e.*, a significantly higher number of females were recovered (63, 70.0%) at the front of the house than at the rear side (*X* ^2^ = 14.4, d.f.: 1, p < 0.001) (Table IV). Moreover, comparison of the total count on the sticky traps showed a similar trend. During the experimental period, 112 (65.9%) females were captured at the front, a significantly higher number than that at the rear side (*X* ^2^ = 17.2, d.f.: 1, p < 0.001). During the entire night, 132 (66.7%) females were captured at the front, a value significantly higher comparison with the total number of females captured at the rear side (*X* ^2^ = 22.1, d.f.: 1, p < 0.001) (Table II).

**TABLE IV:** Numbers of marked blood-fed *Lutzomyia longiflocosa* recaptured at exit points near the eave openings, comparing front of the house to its rear, using sticky traps (nearest zone to the openings, 0-33 cm)

n	Experimental period 21-01 h							Night total 21-06 h					
	
	Front			Rear		Total		Front			Rear		Total
			
	Number (%)			Number (%)				Number (%)			Number (%)		
1	5	(50.0)		5	(50.0)	10		5	(41.7)		7	(58.3)	12
2	5	(62.5)		3	(37.5)	8		8	(61.5)		5	(38.5)	13
3	11	(84.6)		2	(15.4)	13		13	(86.7)		2	(13.3)	15
4	2	(66.7)		1	(33.3)	3		6	(85.7)		1	(14.3)	7
5	18	(72.0)		7	(28.0)	25		20	(71.4)		8	(28.6)	28
6	7	(70.0)		3	(30.0)	10		11	(73.3)		4	(26.7)	15
Total	48	(69.6)		21	(30.4)	69		63	(70.0)		27	(30.0)	90

## DISCUSSION

*Entrance sites* - During the experimental period, the capture of four *Lu. longiflocosa* females on protected human bait inside the modified bedroom suggests that the reduction of the large eave openings (reduced to 5 cm width), owing to the layout of the sticky traps, did not prevent air flow with human kairomones. The sticky traps were efficient in intercepting most of the females attempting to enter the house. Unfed females accounted for 98.0% of the captured phlebotomine sand flies. This result confirms that they were seeking for a human host indoors.

Our results indicate that *Lu. longiflocosa* females tried to enter the house primarily through the large eave openings (width = 10 cm). Slits between the edges of the doors and windows (width = 1 cm) were a secondary entry site. This deduction was based on the following data: (1) a significantly higher number of females were captured at the zone of the sticky trap nearest the eave opening (0-33 cm) than at the other two zones, during the experimental period and all night, and (2) only two females were captured in the cage trap surrounding the slits in the doors and windows during the experimental period and all night.

Only one previous study has shown that sand flies have house entering preferences through large openings. In Marajó Island (Brazil), the size of the openness of the house was positively correlated with the abundance of *Lu. longipalpis* (Quinnell & Dye 1994a). Additional evidence on sand fly preference for large openings comes from two studies conducted on experimental chicken sheds, both also carried out in Brazil. In the first study, most of the individuals of *Lu. longipalpis* were captured in sheds with relatively large openings contrary to close sheds where practically no sand flies were captured (Quinnell & Dye 1994b). Another study found that *Lu. whitmani* numbers increased along with the percentage of shed openings (Campbell-Lendrum et al. 2000). The preference for house entrance through large openings compared with small openings has also been investigated in mosquitoes. A field observation of the houses with open and closed eaves (Lindsay & Snow 1988), and experiments where open eaves were deliberately blocked (Njie et al. 2009), both in Gambia, showed that the number of *An. gambiae* indoors was higher in houses with open eaves than in closed or blocked eaves. Nevertheless, no differences were found in the number of culicines in the latter study. On the other hand, a study in experimental huts with closed eaves, where the door and windows were held ajar (2-3 cm), found no reduction in the indoor density of *An. gambiae* (Lindsay et al. 2003). These results indicate that the house entrance preference of haematophagous Diptera can have variations at interspecific level and according to the availability of the types of openings.

The preference of *Lu. longiflocosa* to enter houses through the large eave openings located at 2.2 m height, compared with the slits in the door and windows, may be explained by two reasons: (1) the absence of obstacles in the large openings, which have been also proposed for mosquitoes (Grieco et al. 2000) and (2) the odor plume with human kairomones which probably rise the house mainly at the eave level, attracting most of the females to this site. Snow (1987) using smoke emanation in an experimental hut showed that smoke came from the downwind and lateral side openings of the hut forming a large plume rising at the eave level. In contrast, in the upwind side of the hut, there was a zone of virtually no wind flow. As the ceiling is located at the eave level, the plume rising from this site may also explain that the percentage of sand flies collected was higher at the ceiling than at the wall.

In relation to the female entrance according to the sides of the house (front versus rear), the significantly higher density of *Lu. longiflocosa* females captured in the trap zone nearest the openings located at the front, compared to the rear side of the house, suggested that females prefer to enter the house through the eave openings located at the front of the house. In other words, the female entry pattern according to the sides of the house is not random.

In other studies, house entering preferences of sand flies in accordance with the sides of the house have not been documented. Although it is recognised that house entry is not random for mosquitoes, little attention has been given to this issue, and information comes mainly from experimental huts. Okumu et al. (2012) observed that the number of mosquitoes entering or exiting a hut through any opposite sides is never equal. Given this non-random entry and exit behaviour, it is recommended to alternate the hut traps regularly (Curtis et al. 1992).

The non-random sand fly entrance to the houses may also be explained by the pattern in which the odor plume leaving the house is transported by air currents; this was similarly explained in the comparison between eave openings and slits. Data on the predominant wind direction during night-time were not recorded in the present study. Nevertheless, the location of the study houses, placed with the front side of the house facing the downslope of the mountain, and the direction of the wind during the night-time (katabatic winds), also downslope (Aranguren et al. 2012), suggest that the downwind side of the house was the front, whereas the upwind side was the rear. Conceivably then, the main odor plume was rising from the eave openings mainly in the front side (downwind) of the houses and thereby attracting most of host seeking females. Phlebotomine sand flies may come to the houses from more than one of the surrounding forests.

In conclusion, in the sub-Andean region of Colombia where most houses are embedded on the mountain facing the valley with the main openings being eave openings and slits between the edges of doors and windows, sand flies enter primarily through the eave openings, whereas slits are secondary entrance sites. Entering behaviour is not random as sand flies prefer to enter through the front side of the houses.

*Exit sites* - Although efforts were made to block openings other than the eave openings and slits in the doors and windows, only 57% of the released females were recaptured in the exit experiment. Nevertheless, the 214 females that were recaptured are considered enough for conclusive results. On the other hand, none of the females released in the modified bedrooms were recaptured with protected human bait. This confirms that the blood-fed females did not represent any risk for the inhabitants of the house or the volunteers during the study period. Long term risks (*e.g.*, bites in subsequent gonotrophic cycles and an eventual increase in sand fly population) of the released blood-fed females which were not recaptured (27 females/trial) were considered negligible. The reasons for this were: (1) blood-fed females used in the experiment came from the same *Lu. longiflocosa* population which currently visit the study houses and (2) the number of females which avoided the sticky traps and returned to the forest were half of the females (50 females/CDC trap/night) which currently enter to get a blood meal in these types of houses (Pardo 2006).

As in the house entering observations, most of the recaptured blood-fed females of *Lu. longiflocosa* attempted or successfully left the house through the large eave openings; only a few females used the slits in the doors and windows. The latter deduction was based on the following data: (1) in the fraction of the sticky trap nearest (0-33 cm) to the large openings at the ceiling, a significantly higher percentage of females was recaptured during the experimental period (86.2%) and all-night collections (79.1%) than the two fractions farther from the openings; (2) nine females (four during the experiment and five in the post-experimental period) were recaptured at the exterior of the houses in the two fractions of the sticky trap in the ceiling located farthest from the openings (33-66 cm and 66-100 cm); and (3) during the experimental period, only seven females were recaptured by the cage traps surrounding the slits in the doors and windows.

The sticky traps on the wall unexpectedly collected the highest percentages of females in the far zone, both during the experimental period and the all-night collections. This might be a consequence of the physical and physiological conditions of the post-fed females and not because the females did not attempt to pass through the eave openings. Immediately after blood-feeding, females probably move with difficulty, because blood-fed females have doubled their normal weight (Ready 1978). Therefore, some blood-fed females released at the centre of the modified bedroom may have first landed on the lower part of the wall, uncovered with sticky trap. From there, females probably started to ascend the wall in shorter flights towards the eave openings and were captured by sticky trap at the far zone (66-100 cm) of the eave openings. The tendency of the blood-fed females to rest on the lower parts of the internal walls was also observed in *Lu. gomezi* in Colombia. A total of 71% of *Lu. gomezi* blood-fed females were captured by sticky traps located at the lowest 100 cm of the walls (Martínez et al. 1995).

Alternatively, it is possible that a number of females that reached the lower part of the wall, immediately after release, continued the process of diuresis in situ (Sadlova et al. 1998). Preliminary observations indicate that diuresis in *Lu. longiflocosa* has a duration of up to 2 h (unpublished observations by the authors). After diuresis, these females may have lost excess weight and recovered their mobility, flying further towards the sticky trap zone nearest the eave opening. In the present study, a high percentage, 85.7% (12/14), of females was recaptured on the wall nearest the large eave openings in the post-experimental period (01-06 h). These females had rested for at least 4 h (during the experimental period) inside the modified bedroom.

To our knowledge, no studies have been performed on sand fly exit behaviour. In the present study, we found a similar behaviour as has been reported for mosquitoes. Several observations have been performed in experimental huts indicating that mosquitoes tend to leave the hut through large openings (Smith 1965, Okumu et al. 2012).

The possible explanation of why *Lu. longiflocosa* prefers the large eave openings to leave the house is in general the same as proposed to explain the entering preferences. The only difference is that in this case, sand flies would not be attracted, but instead repelled from the odor plume. It is possible that the same kairomones and other cues used by sand flies to approach a potential host can be used to move away from it, but in this case by inverting the direction of the orientation mechanism (Lazzari 2009). To our knowledge, no studies have addressed this issue in sand flies.

In relation to the exit preferences according to the sides of the house (front and rear), our results suggest that blood-fed *Lu. longiflocosa*, as observed in the entrance experiment with unfed females, do not leave the houses randomly, preferring the front side instead of the rear side. The latter was based on the greater percentages of captured females at the front of the house than those at the rear side, both during the experimental period and all-night collections, in the sticky trap zone nearest the large eave opening. In mosquitoes, using experimental houses, it has also been found that they do not exit the huts randomly (Okumu et al. 2012).

The behaviour of the sub-Andean endophagic sand fly *Lu. longiflocosa* was clarified in two (close range seeking and initial host leaving) of the three phases of its gonotrophic cycle. With respect to the close range seeking, most of the females preferred to enter through the eave openings at the ceiling level than through the slits between the edges of the doors and windows. Moreover, their preferred entrance with respect to the sides of the house is not random, and the majority of females make contact with the external wall before entering the house. Few females though were able to enter without landing on the walls. In relation with the initial host leaving, blood-fed females leave the house through the same eave openings and with the same non-random pattern used when they enter.

The findings of the present study provide useful information about the entering and exiting behaviour of *Lu. longiflocosa*, which can be used for a more comprehensive evaluation of the impact of the vector control measures against cutaneous leishmaniasis, as well as to envisage new methods or to improve the currently applied. For instance, the high preference of *Lu. longiflocosa* for entering and exiting through the eave openings confirmed that eaves are a valid target for controlling endophagic sand flies. Therefore, the use of netting or curtains to intercept females in the eaves is strongly encouraged. The importance of the external ceiling for house entry must be recognised in the application of insecticides in this type of housing.
